# ^18^F-PSMA-1007 PET/CT for response assessment in patients with metastatic renal cell carcinoma undergoing tyrosine kinase or checkpoint inhibitor therapy: preliminary results

**DOI:** 10.1007/s00259-020-05165-3

**Published:** 2020-12-28

**Authors:** L. M. Mittlmeier, M. Unterrainer, S. Rodler, A. Todica, N. L. Albert, C. Burgard, C. C. Cyran, W. G. Kunz, J. Ricke, P. Bartenstein, C. G. Stief, H. Ilhan, M. Staehler

**Affiliations:** 1 Department of Urology, University Hospital, LMU Munich, Munich, Germany; 2 Department of Nuclear Medicine, University Hospital, LMU Munich, Munich, Germany; 3 Department of Radiology, University Hospital, LMU Munich, Munich, Germany; 4 Head Interdisciplinary Center on Renal Tumors, Department of Urology, University Hospital, LMU Munich, Marchioninistr. 15, 81377 Munich, Germany

**Keywords:** Metastatic renal cell carcinoma, ^18^F-PSMA-1007 PET, CT, Response assessment, Tyrosine kinase therapy, Checkpoint inhibitor therapy

## Abstract

**Introduction:**

Tyrosine kinase (TKI) and checkpoint inhibitors (CI) prolonged overall survival in metastatic renal cell carcinoma (mRCC). Early prediction of treatment response is highly desirable for the individualization of patient management and improvement of therapeutic outcome; however, serum biochemistry is unable to predict therapeutic efficacy. Therefore, we compared ^18^F-PSMA-1007 PET imaging for response assessment in mRCC patients undergoing TKI or CI therapy compared to CT-based response assessment as the current imaging reference standard.

**Methods:**

^18^F-PSMA-1007 PET/CT was performed in mRCC patients prior to initiation of systemic treatment and 8 weeks after therapy initiation. Treatment response was evaluated separately on ^18^F-PSMA-PET and CT. Changes on PSMA-PET (SUV_mean_) were assessed on a per patient basis using a modified PERCIST scoring system. Complete response (CR_PET_) was defined as absence of any uptake in all target lesions on posttreatment PET. Partial response (PR_PET_) was defined as decrease in summed SUV_mean_ of > 30%. The appearance of new, PET-positive lesions or an increase in summed SUV_mean_ of > 30% was defined as progressive disease (PD_PET_). A change in summed SUV_mean_ of ± 30% defined stable disease (SD_PET_). RECIST 1.1 criteria were used for response assessment on CT. Results of radiographic response assessment on PSMA-PET and CT were compared.

**Results:**

Overall, 11 mRCC patients undergoing systemic treatment were included. At baseline PSMA-PET_1_, all mRCC patients showed at least one PSMA-avid lesion. On follow-up PET_2_, 3 patients showed CR_PET_, 3 PR_PET_, 4 SD_PET_, and 1 PD_PET_. According to RECIST 1.1, 1 patient showed PR_CT_, 9 SD_CT_, and 1 PD_CT_. Overall, concordant classifications were found in only 2 cases (2 SD_CT + PET_). Patients with CR_PET_ on PET were classified as 3 SD_CT_ on CT using RECIST 1.1. By contrast, the patient classified as PR_CT_ on CT showed PSMA uptake without major changes during therapy (SD_PET_). However, among 9 patients with SD_CT_ on CT, 3 were classified as CR_PET_, 3 as PR_PET_, 1 as PD_PET_, and only 2 as SD_PET_ on PSMA-PET.

**Conclusion:**

On PSMA-PET, heterogeneous courses were observed during systemic treatment in mRCC patients with highly diverging results compared to RECIST 1.1. In the light of missing biomarkers for early response assessment, PSMA-PET might allow more precise response assessment to systemic treatment, especially in patients classified as SD on CT.

## Introduction

Tyrosine kinase inhibitors (TKIs) and checkpoint inhibitors (CIs) significantly prolong survival in mRCC patients [1–3]. Early prediction of treatment response is highly desirable for individualization of patient management and improvement of outcome. However, established predictive biomarkers for response assessment are lacking [4, 5]. Currently, criteria-based reporting for response assessment relies on morphological imaging criteria such as RECIST 1.1. Unlike most other malignancies, the application of ^18^F-FDG PET/CT in RCC is limited by its low FDG-avidity [6]. Although preliminary data have indicated a potential role of ^18^F-FDG PET/CT for treatment monitoring of nivolumab in RCC patients [7], discordant published data lead to a missing recommendation in current guidelines [8]. PSMA is increasingly recognized in prostate cancer imaging [9]. Moreover, PSMA is highly expressed on the cell surface of the tumor microvasculature of several solid tumors [10, 11]. Initial data showed promising results for PSMA-targeted PET imaging in mRCC and might improve diagnostic accuracy [10, 12–15].

We hypothesized that PSMA expression as a tumoral feature of RCC changes under TKI or CIs therapy and that ^18^F-PSMA-1007 PET provides pathophysiological information beyond morphological extent on CT. We therefore compared ^18^F-PSMA-1007 PET using modified PERCIST criteria to CT response based on RECIST 1.1 in mRCC patients undergoing TKI or CI therapy.

## Methods

### Inclusion criteria

This analysis was approved by the institutional ethics committee of the LMU Munich (IRB# 20-315). Criteria for inclusion were (1) histologically proven mRCC, (2) therapy with TKI or CI, (3) ^18^F-PSMA-1007 PET/CT prior to therapy with TKI or CI, and (4) follow-up ^18^F-PSMA-1007 PET/CT 8 weeks after therapy initiation.

### Radiopharmaceutical and imaging protocol

A median activity of 246 MBq (range 217–268 MBq) ^18^F-PSMA-1007 was injected intravenously in line with previously reported radiosynthesis and administration procedures [16]. Additionally, the patients were premedicated with furosemide (20 mg) if no contraindication was given [17]. The radiopharmaceutical was used on an individual patient basis according to German Pharmaceuticals Act §13(2b). PET was performed from the skull base to the mid-thigh using a Biograph mCT scanner or a Biograph 64 PET/CT scanner (Siemens Healthineers Erlangen, Germany) 60 min after tracer injection. PET/CT included a diagnostic, contrast-enhanced CT scan in the portal–venous phase (Imeron 350; 1.5 ml/kg body weight; Bracco Imaging, Milano, Italy). PET was acquired with 2.5 min per bed position and reconstructed iteratively using TrueX (three iterations, 21 subsets) with Gaussian postreconstruction smoothing (2 mm full-width at half-maximum).

### Radiographic therapy response assessment

Radiographic treatment response was separately assessed on ^18^F-PSMA-1007 PET and CT datasets. For ^18^F-PSMA-1007 PET analysis, images were analyzed independently by two experienced nuclear medicine physicians (MU, HI) on a dedicated workstation (Hermes Hybrid 3D Viewer, Hermes Medical Solutions, Stockholm, Sweden).

#### ^18^F-PSMA-1007 PET

Transaxial PET slices were used for image analysis as described previously [18]. Five organ systems were included per patient comprising lymph nodes, bone, affected kidney/kidney bed, and other visceral metastatic sites. Any focal uptake of ^18^F-PSMA-1007 higher than the surrounding background not associated with physiological uptake was considered suspicious for malignancy. For each organ system, the two lesions with the highest ^18^F-PSMA-1007 uptake were analyzed on PET_1_ (PET_1_ = PET prior to therapy initiation). For quantitative analysis, the slice with the maximum ^18^F-PSMA-1007 was identified using an isocontour volume of interest (VOI) including all voxels above 99% of the maximum covering the whole lesion volume. In a second step, a spherical VOI with a diameter of 1.5 cm was placed over the tumor lesion centering in the slice with the maximum ^18^F-PSMA-1007 uptake, and the mean standardized uptake volume (SUV_mean_) was noted. PET_2_ (PET_2_ = PET 8 weeks after initiation) findings were compared to PET_1_.

Posttreatment changes were interpreted according to modified PET Response Criteria in Solid tumors (PERCIST) 1.0 [18]. The absence of any PSMA-uptake on PET_2_ was defined as molecular complete response (CR_PET_). A decrease in summed SUV_mean_ of ≥ 30% was considered PR_PET_. The appearance of new PET-positive lesions on PET_2_ or an increase in summed SUV_mean_ of ≥ 30% was considered progressive disease (PD_PET_). An intermediate change in summed SUV_mean_ between − 30 and + 30% without new target lesions was considered stable disease (SD_PET_).

#### CT (RECIST 1.1)

For evaluation of CT datasets, response assessment was performed by two experienced radiologists (WGK, CB) according to RECIST 1.1 using a dedicated software (mint lesion™, version 3.0.1, Mint Medical GmbH, Dossenheim, Germany) [18, 19]. Target and nontarget lesions were defined and measured in baseline CT prior to therapy initiation (CT_1_). In the follow-up CT examination 8 weeks after initiation, target lesions were located and manually measured (CT_2_). Disappearance of all lesions was considered complete response (CR_CT_); a decrease in summed diameters of ≥ 30% was defined as partial response (PR_CT_). The appearance of a new target lesion on CT_2_ or an increase in the summed diameters of ≥ 20% with an absolute increase of at least 5 mm was defined as progressive disease (PD_CT_). An intermediate change in summed diameter between − 30% and + 20% without appearance of a new target lesion was considered stable disease (SD_CT_).

### Statistical analysis

Statistical analyses were performed with IBM SPSS® Statistics (version 25, IBM Corp., Armonk, NY). Descriptive statistics are displayed as median (range) or mean ± standard deviation (SD). Relative changes during therapy are displayed as percentage differences.

## Results

### Patients and treatment regimen

Eleven mRCC patients were included in this analysis (mean age 59.6 years (range 24.4–78.4 years; 8 male/3 female). Patients underwent ^18^F-PSMA-1007 PET/CT directly before undergoing therapy with TKI or CI and 8 weeks after therapy initiation. 7/11 (63.6%) patients underwent TKI therapy (2x cabozantinib, 3x sunitinib, 1x axitinib, and 1x levantinib + everolimus), 4/11 (36.4%) patients underwent CI therapy (2x ipilimumab + nivolumab, 1x nivolumab, and 1x pembrolizumab) using standard dosages without dose reduction during follow-up. Baseline characteristics are presented in Table 1.

**Table 1:** Baseline characteristics and comparison between radiographic response on ^18^F-PSMA-1007 and CT

Patient	Age	Sex	Histology	Tumor localization^°^	Therapy	Δ SUVmean (%)	Response on PET	Δ RECIST (%)	Response on CT
1	48.0	F	ccRCC	LN, VO	Cabozantinib	n.e.	CR	− 13.2%	SD
2	77.1	F	ccRCC	K, LN, VO, B	Ipilimumab Nivolumab	− 12.2%	PD^*^	− 29.1%	SD
3	74.8	M	ccRCC	K, LN, VO, B	Levantinib Everolimus	− 28.7%	SD	− 7.2%	SD
4	70.5	M	pRCC	K, LN, VO, B	Sunitinib	− 44.7%	PR	− 1.5%	SD
5	52.9	F	ccRCC	VO	Cabozantinib	n.e.	CR	1.8%	SD
6	70.8	M	ccRCC	VO, LN, B	Sunitinib	− 68.8%	PR	− 18.5%	SD
7	44.9	M	ccRCC	VO	Axitinib	n.e.	CR	− 26.2%	SD
8	24.4	M	pRCC	K, LN, VO	Nivolumab	− 9.9%	SD	24.8%	PD
9	42.8	M	uRCC	K, LN, VO	Ipilimumab Nivolumab	− 14.2%	SD	− 35.5%	PR
10	73.7	M	ccRCC	K, VO, B	Pembrolizumab	− 35.9%	PR	− 18.3%	SD
11	78.4	M	ccRCC	K, VO	Sunitinib	− 28.1%	SD	− 18.9%	SD

°As defined on ^18^F-PSMA-1007. ^*^PD because of new lesions on PET2. *f* female, *m* male, *ccRCC* clear cell renal cell carcinoma, *pRCC* papillary renal cell carcinoma, *uRCC* undifferentiated renal cell carcinoma, *K* kidney, *LN* lymph nodes, *VO* visceral organs, *B* bone, *n.e.* not evaluable, *PD* progressive disease, *PR* partial response, *SD* stable disease

### Response assessment

#### PET-based response assessment

**Fig. 1:**
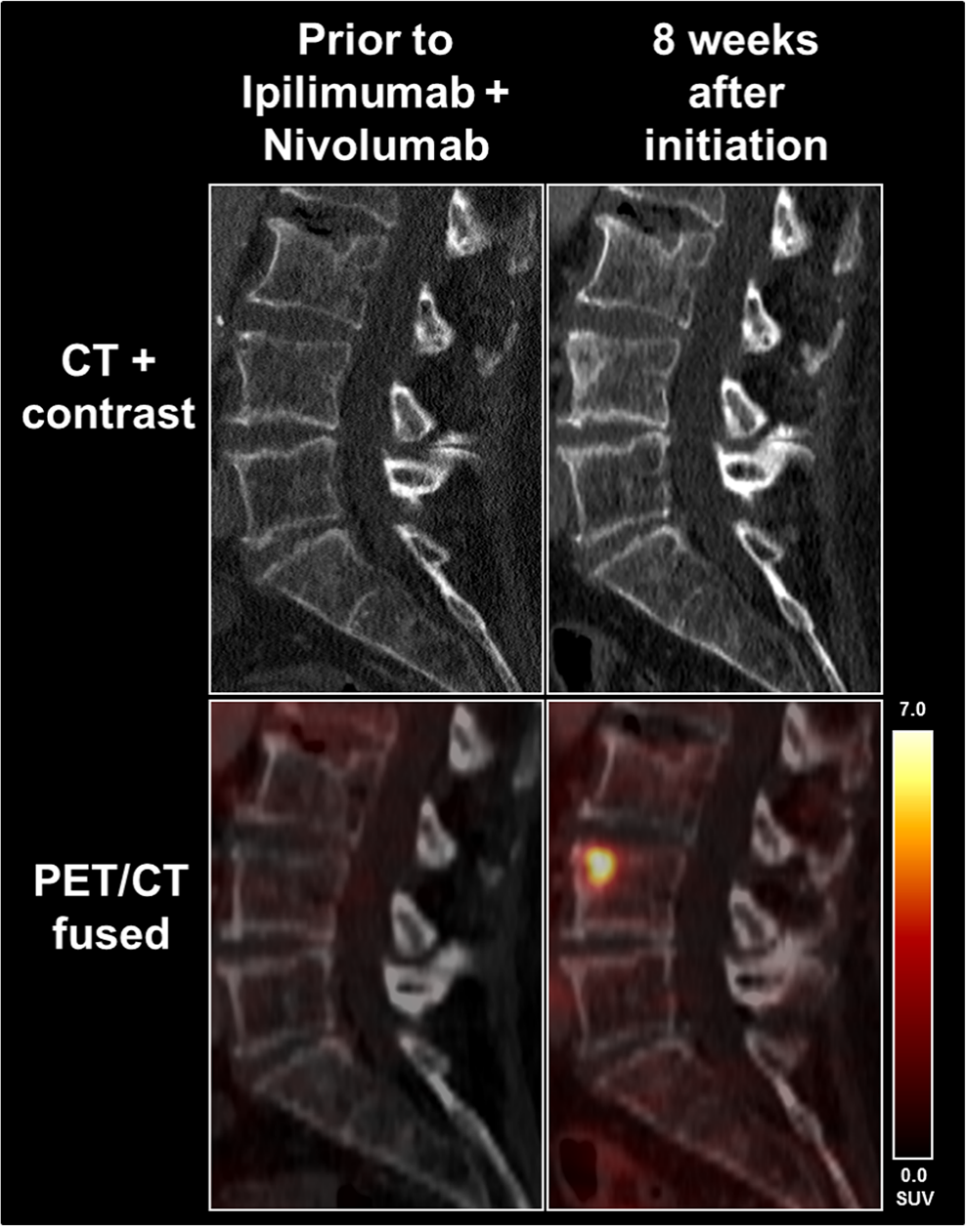
A 77-year-old female patient showed a new osteoblastic lesion on follow-up CT during therapy with Ipilimumab and Nivolumab. According to RECIST 1.1, this is not rated as PD. However, a high PSMA expression could be seen on PET indicating this lesion to be a vital metastasis rather than an avital osteoblastic reaction to therapy. Consequently, this was rated PD_PET_, although the other tumoral lesions showed stable uptake on PET

Three of 11 (27.2%) patients showed CR_PET_ with an absence of any PSMA uptake on PET_2_. Three of 11 (27.2%) showed PR_PET_ with a decrease in summed SUV_mean_ of ≥ 30%; in 4/11 patients (36.4%), an intermediate change in summed SUV_mean_ between − 30% and + 30% without appearance of a new target lesion (SD_PET_) was seen. One of 11 patients (9.1%) presented with a new, PET-positive target lesion and was defined as PD_PET_ (Fig. 1).

#### CT-based response assessment

When analyzing the CT-based response assessment using RECIST 1.1, 1/11 (9.1%) patient showed PR_CT_ with a decrease in summed diameters of ≥ 30% (− 35.5%), 9/11 (81.8%) of the patients showed SD_CT_ with an intermediate change in summed diameter between − 30% and + 20% without appearance of any new target lesion, and 1/11 (9.1%) patients had PD_CT_ with an increase in the summed diameters of ≥ 20% with an absolute increase of at least 5 mm.

#### Concordance of PET- and CT-based response assessment

Overall, concordant results between PET and CT response assessments could only be obtained in 2/11 (18.2%) patients, presenting with SD both on PET and CT (2 SD_CT + PET_). Three patients with CR_PET_ were classified as SD_CT_ on CT, whereas no patient showed CR_CT_.

By contrast, 1 patient classified as PR_CT_ on CT showed PSMA uptake without major changes during therapy (SD_PET_). However, among 9 patients with SD_CT_ on CT, 3 were classified as CR_PET_, 3 as PR_PET,_ 1 as PD_PET_, and only 2 as SD_PET_ on PSMA-PET. Concordance between radiographic responses on PET and CT are presented in Table 2.

**Table 2:** Concordance between radiographic response on PET and CT

Response PET	Response CT				
	Progressive disease	Stable disease	Partial response	Complete response	Total
Progressive disease	0	1*	0	0	1
Stable disease	1°	2	1*	0	4
Partial response	0	3°	0	0	3
Complete response	0	3°	0	0	3
Total	1	9	1	0	11

^*^Better response on CT. °Better response on PET

## Discussion

**Fig. 2:**
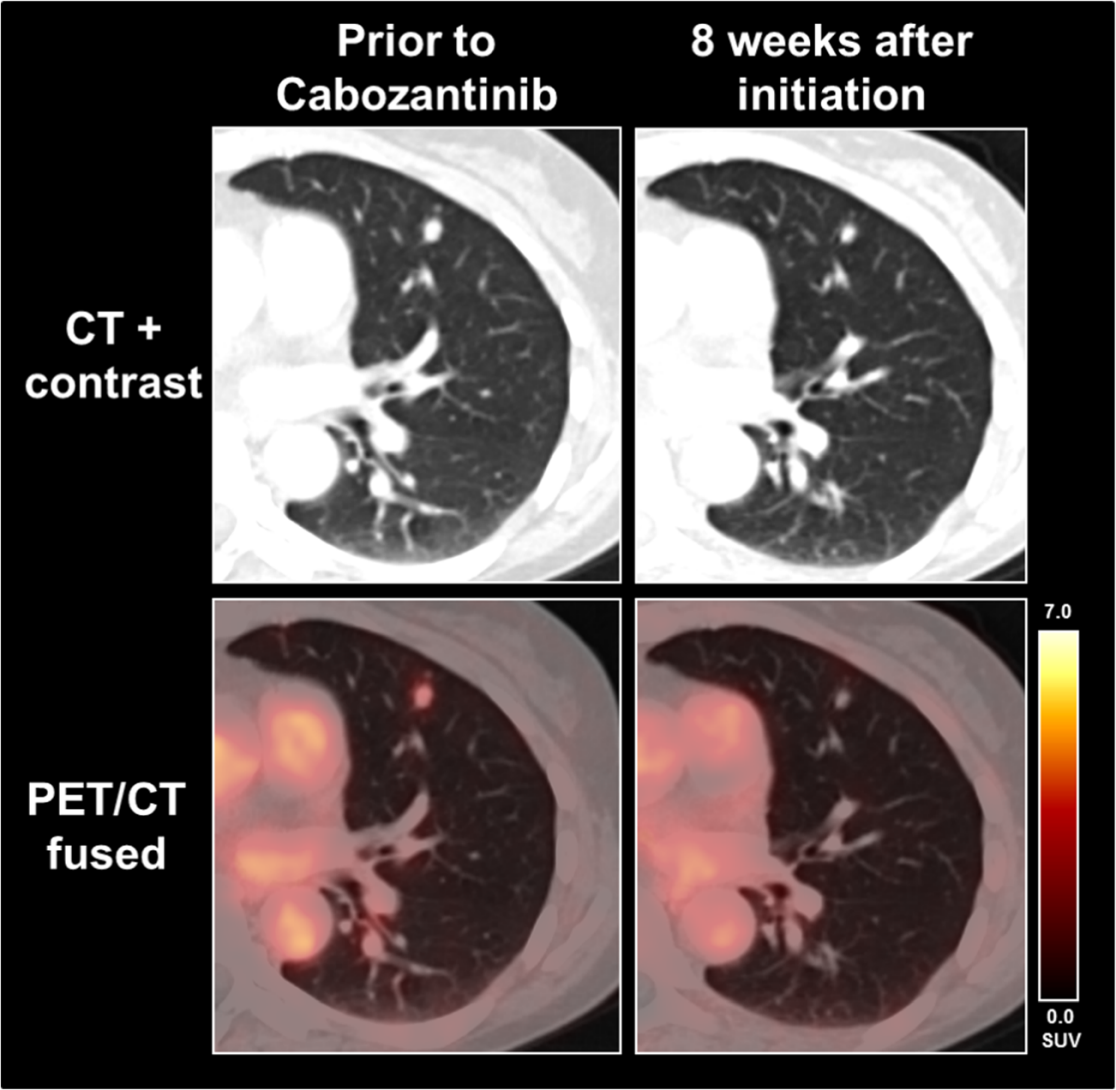
A 53-year-old female patient showed a slightly decreasing pulmonary metastasis, which, however, completely lost PSMA expression during therapy with cabozantinib

Our data demonstrate a change of PSMA-PET expression during systemic therapy of mRCC in the majority of patients; even a complete remission of PSMA-expression was observed in 3/11 patients (27.2%) despite remaining tumor mass with SD on CT (Fig. 2). Interestingly, the evaluated PET response assessment using PERCIST criteria showed vast discrepancies to morphological response assessment using RECIST 1.1. Only 2/11 patients comprised a concordant finding on PET and CT, whereas 9/11 patients (81.8%) showed in parts highly diverging classifications on PSMA-PET and CT. 6/11 patients (54.5%) showed CR or PR on PET and SD using RECIST 1.1. This result suggests that ^18^F-PSMA-1007 PET may be able to assess treatment response on a molecular level earlier than morphological changes on standard imaging (Fig. 2) with potential adjustments of the treatment regimen. These findings underline current data, which could show that PSMA-PET is advantageous over standard imaging with CT alone in mRCC, particularly for the identification of small lesions such as lymph nodes [14]. This additional pathophysiological information beyond CT morphology could also lead to a decision of continuing or changing current therapy or to de-escalate therapy in order to reduce drug-related side effects [3].

Conversely, we also observed changes towards progression on PET with one patient showing PD on PET, but SD on CT. Here, new osteoblastic lesions in vertebra T7 and L4 with focally increased PSMA uptake (Fig. 1) were identified. According to RECIST 1.1, osteoblastic metastases are non-measurable lesions, as they can be seen as a potential sign of treatment response, when changing from lytic to blastic [20]. Therefore, a distinction of vital bone metastases and bone metastases with therapy response remains highly challenging using morphological imaging with CT [21, 22]. Here, PSMA-PET could potentially add relevant clinical information with regard to the response assessment of osseous lesions (Fig. 1).

Also, the scenario of PD on CT, but SD on PET could be observed in the current cohort. It is known that pseudoprogression can occur in patients undergoing immunotherapy [23] leading to an early enlargement of tumor manifestations as part of the treatment effect during the early phases followed by a subsequent shrinkage of tumor lesions [24]. Using RECIST 1.1, this phenomenon would directly lead to the classification of PD. To overcome these limitations of RECIST 1.1, several modified response criteria were suggested. For example, using iRECIST, this phenomenon leads to the classification of immune unconfirmed progressive disease (iUPD) [25], which would lead to an additional earlier follow-up CT scan to confirm either true progression or pseudoprogression during ongoing immunotherapy. In this scenario, ^18^F-PSMA-1007 PET could contribute in the early identification of pseudoprogression and real progression in mRCC patients undergoing immunotherapy.

One major limitation is the small number of patients as well as the retrospective design of the study. According to Seitz et al., we adapted the PERCIST 1.0 criteria [18, 26] for defining the response categories on PSMA-PET. Although this modified approach has been shown to be feasible for PSMA-PET in published studies [18], a prospective validation including endpoints such as overall survival is mandatory to further investigate the use of ^18^F-PSMA-1007 PET for response assessment. Within this process, exact cut-off values on PSMA-PET for the accurate prediction of treatment response in terms of overall survival are yet to be defined. Additionally, new response criteria for immunotherapy monitoring such as ‘PET/CT Criteria for early prediction of Response to Immune checkpoint inhibitor Therapy’ (PECRIT) and ‘PET Response Evaluation Criteria for Immunotherapy’ (PERCIMT) that link RECIST 1.1 and PERCIST 1.0 were recently introduced [27, 28]; these particular specifications of response assessment should also be evaluated in mRCC patients undergoing PSMA-PET/CT and be correlated with the clinical outcome in order to evaluate the best predictive factors on PSMA-PET.

Nonetheless, our preliminary results provide support to the hypothesis that ^18^F-PSMA-1007 PET and its combination with CT provides complementary information on a molecular level for response assessment in mRCC patients undergoing systemic treatment with TKI or CI.

## Conclusion

On PSMA-PET, heterogeneous courses were observed during systemic treatment in mRCC patients with highly diverging results compared to RECIST 1.1 in mRCC patients undergoing systemic treatment with TKI or CI. Hence, hybrid imaging may optimize response assessment of mRCC patients and influence patient management. In the light of missing biomarkers for early response assessment, PSMA-PET might allow more precise response assessment to systemic treatment, especially in those patients classified as stable disease on CT. Data in correlation with clinical outcome parameters are underway.
